# Inflammation markers are associated with frailty in elderly patients with coronary heart disease

**DOI:** 10.18632/aging.101575

**Published:** 2018-10-16

**Authors:** Ping Hou, Hui-Ping Xue, Xin-E Mao, Yong-Nan Li, Lin-Feng Wu, Yong-Bing Liu

**Affiliations:** 1 School of Nursing, Yangzhou University, Yangzhou 225000, China

**Keywords:** frailty, inflammation markers, coronary heart disease, neutrophil to lymphocyte, red blood cell distribution width

## Abstract

The neutrophil-to-lymphocyte ratio (NLR) and red blood cell distribution width (RDW) are important indicators of adverse outcomes and have predictive value for many diseases; however, the relationships between frailty, and the NLR and RDW in patients with coronary heart disease (CHD) have not been determined. In this cross-sectional study, we investigated the association between frailty, and the NLR and RDW in elderly CHD patients ≥ 60 years of age. Frailty was defined according to frailty phenotype. Of 345 patients enrolled in the study, 22.6%, 58.3%, and 19.1% were characterized as robust, pre-frail, and frail, respectively. A significant positive correlation was observed between frailty and the NLR (r = 0.169) and RDW (r = 0.196). After adjusting for confounders, linear regression analyses showed that participants in the 4th quartile of the NLR or RDW were more likely to have a higher frailty phenotype score. Based on multivariable logistic regression, patients in the 4th quartile of the NLR and RDW, the fully-adjusted odds ratios for incident frailty were 2.894 (p = 0.011) and 2.494 (p = 0.040), respectively. Our findings indicate that frailty is associated with the NLR and RDW in elderly patients with CHD.

## Introduction

Frailty is a prevalent geriatric syndrome characterized by an age-related cumulative decline of the physiologic reserve capacity of multiple systems, which leads to a decreased ability to cope with stress among older adults [1]. Frailty can increase the risk of adverse health outcomes, such as falls, institutionalization, disability, and mortality [2,3]. Coronary heart disease (CHD) is one of the most common diseases in the elderly. Frailty is closely related to the prognosis of CHD. Compared with non-frail patients, the mortality rate of frail, CHD patients is significantly increased and hospital stays are longer [4–6]. Frailty is also an independent predictor of major bleeding and has a negative impact on the quality of life in elderly patients with acute coronary syndrome (ACS) [7,8].

Several studies have demonstrated that chronic systemic inflammation is the underlying etiology of frailty [9,10]. The neutrophil-to-lymphocyte ratio (NLR), a novel marker of inflammation which contains two types of leukocyte subtypes, reflects the balance between neutrophil and lymphocyte levels, and is more accurate than neutrophil to lymphocyte [11]. The NLR is associated with all-cause mortality, the severity and complexity of diseases, and can predict adverse events in patients with stable angina (SA) and ACS [12,13]. The NLR can also forecast the occurrence of renal complications and cardiovascular events in diabetic patients [14]. Compared with healthy people, the NLR is higher in patients with rheumatoid arthritis, Behçet’s disease, ankylosing spondylitis [15]. An increased NLR was recently shown to be associated with the incidence of frailty and survival outcomes in cancer patients [16].

The red blood cell distribution width (RDW) is a parameter reflecting the heterogeneity of red blood cells in the blood and is also a new inflammatory marker [17]. A number of studies have indicated that an increased RDW, as an important indicator of adverse outcomes, has predictive value for the prognosis of patients with cardiovascular disorders, sepsis, and hematologic malignancies [18–20]. Another study showed that a RDW > 14% increases the risk of metabolic syndrome and long-term all-cause mortality [21]. One study reported a correlation between higher values of the RDW and a higher risk of frailty in community-dwelling older adults [22].

The NLR and RDW, as easily detected, highly reproducible, and low-cost inflammatory markers, reflect the status of inflammatory diseases; however, few studies have determined the relationship between frailty, and the NLR and RDW [16,22]. Indeed, this relationship in patients with CHD has not been investigated. We hypothesize that the NLR and RDW levels have independent associations with the incidence of frailty in CHD patients. To investigate such associations will help understand the mechanisms underlying frailty and facilitate more effective management of CHD patients with frailty, reduce hospital stays and all-cause mortality, and improve the quality of life.

## RESULTS

### General characteristics of elderly patients

Three hundred seventy-five patients were selected by simple random sampling, and 30 patients declined to participate this research after explanation. Finally, a total of 345 patients were recruited, making response rate 92.0%; there were no missing data.

**Figure 1:**
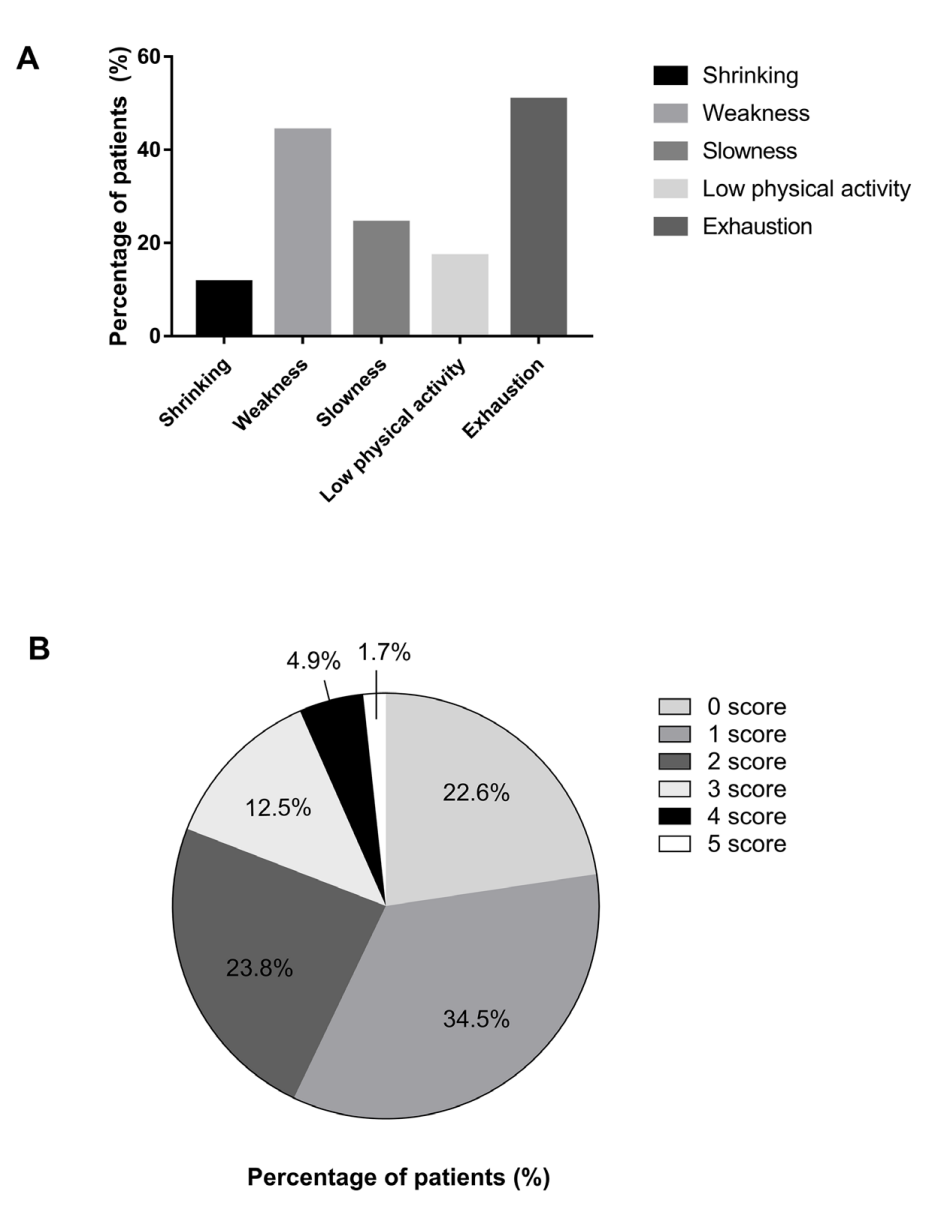
**Detailed distribution of five frailty indicators and frailty phenotype scores.** (**A**) Detailed distribution of five frailty indicators. (**B**) Detailed distribution of frailty phenotype scores.

Three hundred forty-five participants were categorized into the following three groups based on frailty phenotypes: robust (22.6%); pre-frail (58.3%); and frail (19.1%). A detailed distribution of five frailty indicators is demonstrated in Figure 1. The general characteristics of patients are presented in Table 1. The median age was 71.0 years (IQR, 65.0 - 77.0 years). Females accounted for 44.6% of the participants. The median BMI was 24.1 kg/m^2^ (IQR, 22.2 - 26.7 kg/m^2^). Of the patients, 31.3% had a senior middle school or above education. Histories of ACS and single-vessel disease were present in 83.8% and 66.4% of the patients, respectively. The median NLR was 2.6 (IQR, 1.8 - 3.6) and the median RDW was 12.9% (IQR, 12.5 - 13.5%). Age, BMI, hypertension, diabetes mellitus, the NLR, the RDW, the neutrophil, lymphocyte, and erythrocyte counts, and the hemoglobin concentration were significantly different among the three groups. There was no statistical difference in gender, level of education, types of CHD, severity of coronary artery disease, hyperlipidemia, smoking, consumption of alcohol, and leukocyte, monocyte, and platelet counts.

**Table 1:** Clinical characteristics of the 345 study participants according to frailty status.

**Characteristics**	**Robust (n=78)**	**Prefrail (n=201)**	**Frail (n=66)**	**p value**
Age (years, median [IQR])	66.0 (64.0-71.0)	72.0 (65.5-77.0)	76.0 (69.8-81.3)	<0.001
Gender (female, %)	30 (38.5%)	91 (45.3%)	33 (50.0%)	0.367
BMI (median [IQR])	25.0 (23.2-26.9)	24.0 (22.2-26.8)	23.3 (20.8-25.6)	0.007
Education level (less than high school, %)	48 (61.5%)	137 (68.2%)	52 (78.8%)	0.082
Types of CHD (ACS, %)	64 (82.1%)	166 (82.6%)	59 (89.4%)	0.385
Coronary arteries severity (Single-vessel disease, %)	54 (69.2%)	138 (68.7%)	37 (56.1%)	0.142
Hypertension (n, %)	10 (12.8%)	65 (32.3%)	16 (24.2%)	0.004
Diabetes mellitus (n, %)	59 (75.6%)	155 (77.1%)	39 (59.1%)	0.014
Hyperlipidemia (n, %)	51 (65.4%)	152 (75.6%)	53 (80.3%)	0.097
Smoking (n, %)	46 (59.0%)	127 (63.2%)	37 (56.1%)	0.546
Consumption of alcohol (n, %)	56 (71.8%)	155 (77.1%)	52 (78.8%)	0.557
NLR (median [IQR])	2.3 (1.7-3.1)	2.6 (1.9-3.6)	2.9 (2.3-4.2)	<0.001
RDW, % (median [IQR])	12.8 (12.4-13.2)	12.9 (12.5-13.5)	13.1 (12.7-14.2)	0.002
Leukocyte, ×10^9^/L (median [IQR])	5.9 (5.0-6.9)	5.9 (4.7-7.2)	6.4 (5.3-7.4)	0.080
Neutrophil, ×10^9^/L (median [IQR])	3.5 (3.0-4.3)	3.9 (2.9-4.8)	4.4 (3.5-5.2)	0.005
Lymphocyte, ×10^9^/L (median [IQR])	1.7 (1.3-1.9)	1.4 (1.1-1.9)	1.3 (1.1-1.8)	0.022
Monocyte, ×10^9^/L (median [IQR])	0.4 (0.3-0.5)	0.4 (0.3-0.5)	0.4 (0.3-0.5)	0.595
Erythrocyte, ×10^12^/L (median [IQR])	4.4 (4.2-4.8)	4.4 (3.9-4.8)	4.2 (3.9-4.6)	0.002
Hemoglobin, g/L (median [IQR])	137.5 (129.8-148.0)	134.0 (123.0-144.5)	129.0 (117.3-137.0)	<0.001
Platelet, ×10^9^/L (median [IQR])	182.5 (149.5-212.3)	168.0 (142.0-202.0)	173.5 (145.3-231.5)	0.192

Abbreviations: IQR, interquartile range; BMI, body mass index; CHD, coronary heart disease; ACS, acute coronary syndrome; NLR, neutrophil lymphocyte ratio; RDW, red blood cell distribution width.

### Correlation between frailty and inflammation markers

Table 2 shows the correlation between inflammatory markers and frailty. A significant positive correlation was observed between the frailty phenotype score and the NLR (r = 0.169, p = 0.002), RDW (r = 0.196, p < 0.001). We also evaluated the correlation between inflammatory factors and five frailty indicators. A positive correlation was also noted between the NLR, and weakness (r = 0.211, p < 0.001) and slowness (r = 0.176, p = 0.001). A significant positive correlation existed between the RDW, and shrinking (r = 0.128, p = 0.018), weakness (r = 0.178, p = 0.001), low physical activity (r = 0.108, p = 0.045), and exhaustion (r = 0.106, p = 0.049).

**Table 2:** The relationship between frailty and inflammatory markers.

**Frailty items**	**NLR**		**RDW**	
	**r**	**p value**	**r**	**p value**
Frailty phenotype score	0.169	0.002	0.196	<0.001
Shrinking	-0.041	0.445	0.128	0.018
Weakness	0.211	<0.001	0.178	0.001
Slowness	0.176	0.001	0.063	0.241
Low physical activity	0.104	0.054	0.108	0.045
Exhaustion	0.038	0.478	0.106	0.049

Abbreviations: r, Spearman correlation coefficient; NLR, neutrophil lymphocyte ratio; RDW, red blood cell distribution width.

### Association between frailty and inflammation markers

The NLR and RDW were taken quartiles for linear regression analyses and multiple regression analyses. NLR levels are as follows: 1st quartile (≤1.8), 2nd quartile (1.9-2.6), 3rd quartile (2.7-3.6), 4th quartile (>3.6). RDW levels are as follows: 1st quartile (≤12.5%), 2nd quartile (12.6%-12.9%), 3rd quartile (13.0%-13.5%), 4th quartile (>13.5%). Linear regression analyses of the frailty phenotype score with the NLR and RDW quartiles are presented in Table 3. Model 1 shows that the frailty phenotype score is associated with the NLR (β= 0.769, p < 0.001) and RDW (β= 0.698, p < 0.001). Model 2, after adjusting for age and gender, shows that inflammation markers are associated with the frailty phenotype score (NLR: β= 0.638, p < 0.001; RDW: β= 0.511, p = 0.004). Model 3, after adjusting for age, gender, education level, BMI, types of CHD, coronary artery disease severity, hypertension, diabetes mellitus, hyperlipidemia, smoking, and consumption of alcohol, showed that participants in the 4th quartile of the NLR or RDW were more likely to have a higher frailty phenotype score (NLR: β= 0.603, p < 0.001; RDW: β= 0.499, p = 0.004). Table 4 shows the crude and adjusted odds ratios of incident frailty/pre-frailty according to the NLR and RDW quartiles. After adjusting the above-mentioned 11 confounders in linear regression analyses, patients in the NLR or RDW 4th quartile increased the risk for frailty/pre-frailty (NLR: OR = 2.894, 95% CI = 1.280 - 6.541, p = 0.011; RDW: OR = 2.494, 95% CI = 1.006 - 6.181, p = 0.048).

**Table 3:** Linear regression analyses between frailty and inflammatory markers.

	**Model 1**			**Model 2**			**Model 3**		
	**β**	**95% CI**	**p value**	**β**	**95% CI**	**p value**	**β**	**95% CI**	**p value**
NLR^a^									
1st	1			1			1		
2nd	0.228	-0.121 - 0.577	0.200	0.141	-0.190 - 0.473	0.403	0.121	-0.206 - 0.449	0.466
3rd	0.237	-0.119 - 0.593	0.191	0.139	-0.200 - 0.477	0.420	0.140	-0.193 - 0.474	0.408
4th	0.769	0.415 - 1.123	<0.001	0.638	0.298 - 0.978	<0.001	0.603	0.268 - 0.939	<0.001
RDW^b^									
1st	1			1			1		
2nd	0.160	-0.190 - 0.509	0.369	0.118	-0.220 - 0.456	0.493	0.218	-0.118 - 0.554	0.204
3rd	0.132	-0.211 - 0.475	0.450	0.106	-0.222 - 0.434	0.525	0.156	-0.165 - 0.477	0.341
4th	0.698	0.341 - 1.055	<0.001	0.511	0.164 - 0.857	0.004	0.499	0.156 - 0.842	0.004

Model 1: Unadjusted; Model 2: Adjusted age and gender; Model 3: Adjusted age, gender, education level, BMI, types of CHD, coronary arteries severity, hypertension, diabetes mellitus, hyperlipidemia, smoking, consumption of alcohol. Abbreviations: β, regression coefficient; NLR, neutrophil lymphocyte ratio; RDW, red blood cell distribution width. a NLR levels are as follows: 1st quartile (≤1.8), 2nd quartile (1.9-2.6), 3rd quartile (2.7-3.6), 4th quartile (>3.6). b RDW (%) levels are as follows: 1st quartile (≤12.5), 2nd quartile (12.6-12.9), 3rd quartile (13.0-13.5), 4th quartile (>13.5)

**Table 4:** Multiple regression analyses between frailty and inflammatory markers.

	**Model 1**			**Model 2**			**Model 3**		
	**OR**	**95% CI**	***p* value**	**OR**	**95% CI**	***p* value**	**OR**	**95% CI**	***p* value**
NLR^a^									
1st	1			1			1		
2nd	1.937	0.998 - 3.760	0.051	1.710	0.853 - 3.427	0.130	1.699	0.820 - 3.519	0.154
3rd	2.510	1.233 - 5.108	0.011	2.267	1.078 - 4.766	0.031	2.528	1.162 - 5.500	0.019
4th	3.368	1.588 - 7.142	0.002	2.762	1.261 - 6.048	0.011	2.894	1.280 - 6.541	0.011
RDW^b^									
1st	1			1			1		
2nd	1.251	0.663 - 2.474	0.520	1.142	0.555 - 2.352	0.718	1.052	0.495 - 2.233	0.896
3rd	1.006	0.525 - 1.926	0.986	0.936	0.474 - 1.847	0.848	1.035	0.511 - 2.095	0.925
4th	2.806	1.226 - 6.424	0.015	2.081	0.880 - 4.920	0.095	2.494	1.006 - 6.181	0.048

Model 1: Unadjusted; Model 2: Adjusted age and gender; Model 3: Adjusted age, gender, education level, BMI, types of CHD, coronary arteries severity, hypertension, diabetes mellitus, hyperlipidemia, smoking, consumption of alcohol. Abbreviations: OR, odds ratio; NLR, neutrophil lymphocyte ratio; RDW, red blood cell distribution width. ^a^ NLR levels are as follows: 1st quartile (≤1.8), 2nd quartile (1.9-2.6), 3rd quartile (2.7-3.6), 4th quartile (>3.6). ^b^ RDW (%) levels are as follows: 1st quartile (≤12.5), 2nd quartile (12.6-12.9), 3rd quartile (13.0-13.5), 4th quartile (>13.5)

## DISCUSSION

In this cross-sectional study of 345 elderly participants referred to the Department of Cardiology at the Affiliated Hospital of Yangzhou University, we investigated the relationship between inflammatory markers and frailty in CHD patients. The results suggested that frailty is associated with the NLR and RDW. Specifically, an increased NLR and RDW was shown to result in a corresponding increase in the risk of frailty. Adjusting confounding factors did not change the association between the NLR and RDW with frailty.

Few studies have examined the association between the NLR and frailty. As a novel inflammatory factor, the mechanism by which NLR acts on frailty has not been fully investigated, and based on our literature search, only one study has focused on the NLR and frailty. Nishijima et al. [16] conducted a cross-sectional study involving cancer patients ≥ 65 years of age to evaluate the relationship between the NLR and frailty. The 36-item Carolina Frailty Index was used to define frailty status [23]. Nishijima et al. [16] reported that the NLR is positively correlated with frailty (r = 0.220, p = 0.025). Participants in the top tertile (> 4.2) of the NLR had a near 4-fold increased risk for frailty compared with people in the bottom tertile (< 2.5) of the NLR (p = 0.031). Our results agree with the results of the Nishijima et al. [16] study. Another study explored the value of the neutrophil and lymphocyte counts in elderly females who were institutionalized in long-stay centers [24]. The results demonstrated that the neutrophil count was positively correlated with the frailty phenotype score (r = 0.380, p < 0.050) and the lymphocyte count was negatively correlated with the frailty phenotype score (r = -0.370, p < 0.050). A greater grip strength was associated with an increased lymphocyte count (r = 0.380, p < 0.050). In our study, a higher NLR was positively correlated with the frailty phenotype (r = 0.169, p = 0.002) and low grip strength (r = 0.211, p < 0.001). Frailty was associated with a change in the inflammatory state. Increased inflammatory factors are important factors with respect to the incidence of frailty [25,26]. The NLR is a significant indicator of inflammation. Inflammatory reactions lead to ischemic damage, the concentration of neutrophils and monocytes in the peripheral blood is increased, and the lymphocyte concentration is reduced to improve local hypoxia [27]. The NLR has been shown to be an effective predictor of prognosis for many diseases. One study reported that the pre-operative NLR is related to the prognosis of many types of cancer and an elevated NLR is associated with poor survival [28]. A meta-analysis involving 23 studies with > 16,000 patients with ACS assessed the effect of NLR on clinically important outcomes and showed that an elevated NLR on admission to the hospital increased the risk of major adverse cardiovascular events, and in-hospital and long-term mortality [29]. Among patients who undergo percutaneous coronary intervention, the NLR is a prognostic marker [30]. An increased NLR is also related to complex coronary arteries [31].

There is limited research pertaining to frailty and RDW at present. A cross-section, population-based study explored the relationship between the RDW and frailty phenotype in 255 community-dwelling adults ≥ 65 years of age. The results showed that higher values of the RDW are associated with the frailty phenotype (OR = 2.15, 95% CI = 1.263–3.683, p=0.019) [22]. We found that participants in the 4th quartile of the RDW were at increased risk of being frail/pre-frail (OR = 2.494, 95% CI = 1.006 - 6.181, p = 0.048) because the RDW is increased in frail, elderly patients with CHD for several reasons. First, the etiology underlying frailty is inflammation. Inflammation can increase of red blood cell clearance, inhibit erythropoietin, reduced iron availability and raise the RDW [32,33]. Some studies have shown that the RDW is also significantly associated with other inflammatory factors, such as C-reactive protein, the erythrocyte sedimentation rate, tumor necrosis factor-α, and interleukin-6 [34,35]. Second, malnutrition plays an important role in frailty [36]. We observed that the BMI was decreased in the frailty group; specifically, the participants in the frail group had the lowest BMI. Loss of appetite in elderly patients decreases the intake of iron, folic acid, and vitamin B, which are vital to erythrocyte maturation. RDW is a prognostic factor for many diseases and can predict all causes of death [37,38]; however, the underlying mechanism and function in frailty warrant further research.

There were some limitations in this study. First, the sample size was relatively small. ACS accounts for the majority of CHD types and most patients have single-vessel disease, although we did not detect a statistical difference among the three groups according to frailty status. Because of the small number of patients, we failed to analyze the relationship between inflammatory factors and frailty in the different sub-groups (ACS vs. SA and single-vessel disease vs. multi-vessel disease). In a corollary study, we will increase the sample size and analyze the levels of inflammatory factors in various sub-groups to better explore the mechanism underlying frailty. Second, in this cross-sectional study, we unable to assess the causal relationship between frailty and inflammatory markers. In the future, longitudinal studies will be designed to explore the predictive effects on frailty. Third, even though we excluded patients with acute infections, autoimmune diseases, blood systemic diseases, malignancies, and thyroid disorders, we could not eliminate other potential infections or ineffective erythropoiesis that may have increased or decreased the NLR or RDW. Fourthly, because the research population was only comprised of elderly Chinese patients who had CHD, therefore selected outcomes may not be extrapolated to other countries. Despite these limitations, the study had several significant advantages. First, this was the first study to investigate the relationship between inflammatory markers (NLR and RDW) and frailty in CHD patients. Coronary artery stenosis was assessed by the gold standard technique (coronary angiography). Finally, the results of this study support the original hypothesis and suggest a potential role for the NLR and RDW in the pathogenesis of frailty in patients with CHD.

To summarize, an increased NLR or RDW was associated with a corresponding increased risk for frailty. Currently, routine blood testing is standard for every CHD patient. As simple, effective, economic inflammatory markers, the NLR and RDW deserve more attention by clinicians to identify and manage frailty patients in a timely fashion, reduce adverse outcomes and medical expenses, and improve the quality of life.

## MATERIALS AND METHODS

### Study design

A cross-sectional study was conducted to assess the relationship between frailty and NLR, RDW in patients with CHD.

### Study population

We recruited patients by simple random sampling (the random number method) from May 2017 to May 2018 in Department of Cardiology of Affiliated Hospital of Yangzhou University. Sample size was determined by 66.7% proportion of prefrail and frail [39], with a 95% confidence level and 5% margin of error. By adding 10% non-response rate, the total sample size was 375.

We selected elderly patients > 60 years of age with CHD. The diagnostic criterion for CHD, based on coronary angiography, was the presence of at least one coronary artery with > 50% severe strictures. Exclusion criteria were as follows: (1) acute infections, autoimmune diseases, blood systemic diseases, malignancies, thyroid disorders, congestive heart failure, heart valve disease, and liver or kidney dysfunction; (2) medications which can increase or decrease red blood cells, immunosuppressants, or oral corticosteroids in the last 3 months; (3) a history of radiotherapy or chemotherapy; (4) a surgical procedure within 3 months; (5) a history of a digestive tract hemorrhage, blood transfusion, or blood donation in the last 6 months; (6) long-term bedrest or a fracture that interferes with the measurement of grip strength and pace; (7) blood samples could not collected within 24 h after admission; and (8) declined to participate in the study or had missing data. Ethical approval was obtained from the Ethics Committee of the Medical College of Yangzhou University. All clinical investigations were conducted according to the principles expressed in the Declaration of Helsinki. Written informed consent was obtained from all participants. All personal information was encrypted.

### Frailty phenotype

The frailty phenotype, which consists of five frailty indicators, was used to assess frailty, as follows [3]: (1) Shrinking is an unintentional loss of ≥ 4.5 kg or a loss of ≥ 5% body weight in the past 1 year. (2) A hydraulic dynamometer was used to measure grip strength as an indicator of weakness. Older adults in a sitting position used the dominant hand to grip an object 3 times and the researcher recorded the maximum value. We used the standards proposed by Fried et al. [3] to define low grip strength. (3) The time required to walk 4.6 meters at a normal speed was used as an indicator of slowness. Slow walking speed was defined as ≥ 6 seconds when a male is > 173 cm in height and a female is > 159 cm in height or 7 seconds when a male is ≤ 173 cm in height and a female is ≤159 cm in height. (4) The International Physical Activity Questionnaire was used to assess physical activity [40]. Males who expended < 383 kcal/w and females who expended < 270 kcal/w were considered to have low physical activity. (5) The following two questions from the CES-D were used to assess poor endurance and energy [41]. ‘In the last week, I felt that everything I did was an effort’ and ‘Could not get going in the last week.’ If positive response was given to either of these questions, the participant was thought to be exhausted. Individuals with 0, 1-2, and > 3 frailty indicators were categorized as robust, pre-frail, and frail, respectively.

### Correlation variable

Correlation variables included the following: (1) social demographic data (age, gender, and level of education); (2) health status (body mass index [BMI], types of CHD, severity of coronary artery disease, hypertension, diabetes mellitus, and hyperlipidemia); and (3) unhealthy behaviors (smoking cigarettes and consuming alcohol). Patients wore lightweight clothing without hats and shoes to measure the height and weight. The BMI was calculated as the weight (kg)/height (m)^2^. CHD was divided into two categories (ACS and SA). According to a self-reported history of diabetes mellitus or use of anti-diabetic medications, hypertension or use of anti-hypertensives, and hypercholesterolemia or use of a statin or lipid-lowering medication, the participants were considered to have diabetes mellitus, hypertension, and hyperlipidemia, respectively. Participants who smoked ≥ 100 cigarettes in their lifetime were considered to be smokers. An amount of alcohol in excess of 25 g (male) or 15 g (female) per day was defined as alcohol consumption.

### Inflammatory markers

Fasting blood was exsanguinated from the vein by medical professionals within 24 h after admission. Blood samples were then sent to the Clinical Laboratory at the Affiliated Hospital of Yangzhou University. The NLR and RDW were measured on automated instruments.

Before the investigation, the researchers were trained for 1 week to ensure that all the staff utilized uniform methods, followed consistent diagnostic criteria and standardized operation. For patients who considered it inconvenient to complete the questionnaire, the researchers asked the patients each item and recorded the answers. After the questionnaire completed, the researchers immediately conducted a comprehensive inspection to assure that there were no unfilled items. The information was inputted by two researchers, and the third researcher spot checked the inputted information.

### Statistical analysis

Patients were recruited by simple random sampling and divided into three groups according to frailty phenotype. After a normality test, we used the median and interquartile range (IQR) to describe continuous variables that were not normally distributed with assessment by the Kruskal-Wallis test. Categorical variables are shown as frequencies and were assessed by a chi-square test. The correlations between the NLR and RDW, and frailty phenotype score and the five frailty indicators were examined using Spearman’s rho correlation coefficients. Linear regression was performed to determine the association between inflammatory factors and the frailty phenotype score. Potential confounders (age, gender, level of education, BMI, types of CHD, severity of coronary artery disease, hypertension, diabetes mellitus, hyperlipidemia, cigarette smoking, and consumption of alcohol) were adjusted in multiple linear regression. Multivariable logistic regression models after adjusting for potential confounders were performed to evaluate the effects of each inflammatory factor on frailty (robust versus pre-frail or frail). Because inflammatory factors were not normally distributed, we took quartiles of the NLR and RDW for linear regression analyses and multiple regression analyses. P-values < 0.05 were considered statistically significant. All statistical analyses were conducted using SPSS 21.0 (SPSS Inc., Chicago, IL, USA).
